# Rapid response to fifth-line brigatinib plus entrectinib in an *ALK*-rearranged lung adenocarcinoma with an acquired *ETV6*-*NTRK3* fusion: a case report

**DOI:** 10.3389/fonc.2024.1339511

**Published:** 2024-04-18

**Authors:** Dan Li, Yue Zhu, Jincheng Song, Dafu Yang, Saiqiong Cui, Xin Liu, Le Wang, Jiangyan Zhang, Evenki Pan, Zhaoxia Dai

**Affiliations:** ^1^ Department of Medical Oncology, The Second Hospital of Dalian Medical University, Dalian, China; ^2^ Department of Medical Services, Nanjing Geneseeq Technology Inc., Nanjing, Jiangsu, China

**Keywords:** *ALK* rearrangement, brigatinib, entrectinib, resistance mutations, *ETV6-NTRK3*

## Abstract

The management of non-small cell lung cancer (NSCLC), specifically targeting the anaplastic lymphoma kinase (*ALK*) with tyrosine kinase inhibitors (TKIs), is challenged by the emergence of therapeutic resistance. Resistance mechanisms to *ALK* TKIs can be broadly classified into ALK-dependent and ALK-independent pathways. Here, we present a case with lung adenocarcinoma (LUAD) harboring an *ALK* rearrangement. The patient had developed resistance to sequential ALK TKI therapies, with an acquired *ETV6-NTRK3* (E4:N14) fusion as a potential mechanism of *ALK*-independent resistance to lorlatinib. Subsequently, the patient was treated with the combination of brigatinib plus entrectinib and demonstrated a positive response, achieving an 8-month progression-free survival. Our case provides a potential treatment option for LUAD patients with *ALK* rearrangements and highlights the utility of next-generation sequencing (NGS) in uncovering genetic alterations that can guide the selection of effective treatment strategies.

## Introduction

Tyrosine kinase inhibitors (TKIs) targeting driver alterations have been an established modality in treating non-small cell lung cancer (NSCLC) (1). However, the duration of response to TKIs was often limited by acquired drug resistance (2). Resistance to treatment in anaplastic lymphoma kinase (*ALK*)-rearranged NSCLC involve both ALK-dependent and ALK-independent mechanisms (3). ALK-dependent resistance typically arises from secondary mutations within the ALK tyrosine kinase domain. On the other hand, ALK-independent resistance is often due to the activation of bypass signaling pathways, highlighting the complexity and challenges in the management of resistance. Notably, oncogenic alterations in bypass signaling pathways, such as mutations in the epidermal growth factor receptor signaling pathway have been reported (3, 4). In addition, a rare genetic abnormality involving the neurotrophic tyrosine receptor kinase 3 (*NTRK3*) gene fusion has recently been identified in an *ALK*-rearranged NSCLC patient following lorlatinib treatment (5). It is noteworthy that genetic testing was not performed prior to the initiation of ALK inhibitor treatment in the case, so it is unclear whether the *NTRK3* fusion was primary or therapy-induced. In this case report, we delve into the clinical consequences of the *ETV6-NTRK3* (E4:N14) fusion as a resistance mechanism and evaluate the therapeutic potential of a combination treatment with brigatinib and entrectinib in a heavily pre-treated *ALK*-rearranged NSCLC patient.

## Case presentation

**Figure 1:**
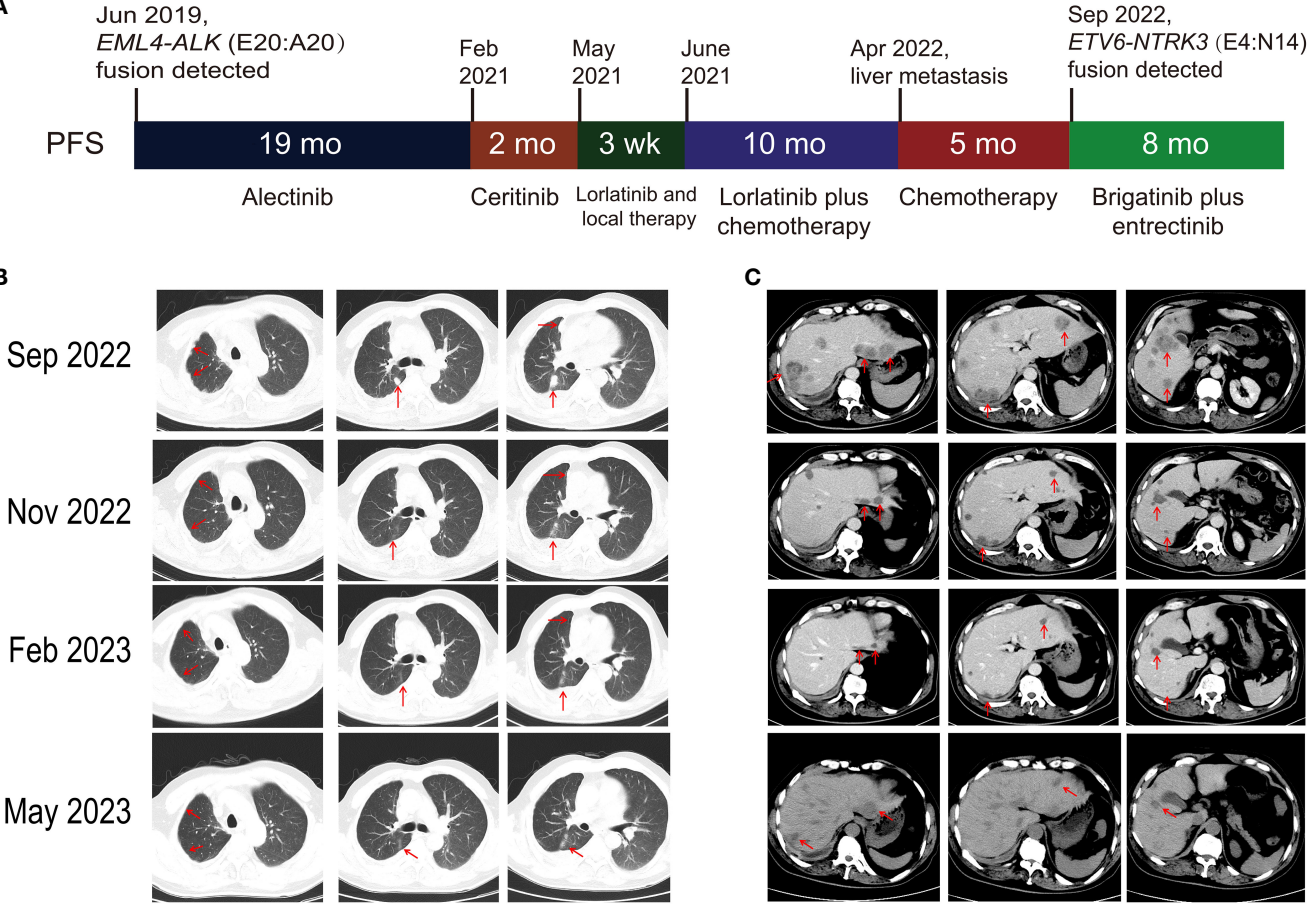
Patient’s clinical progression and radiologic response to brigatinib plus entrectinib therapy. **(A)** Schematic overview of clinical progression. **(B)** Pulmonary and **(C)** liver computed tomography findings: pre-treatment (Sep 2022) and 2 months post-treatment (Nov 2022), 5 months post-treatment (Feb 2023), and 8 months post-treatment (May 2023) with brigatinib plus entrectinib. PFS, progression-free survival.

**Figure 2:**
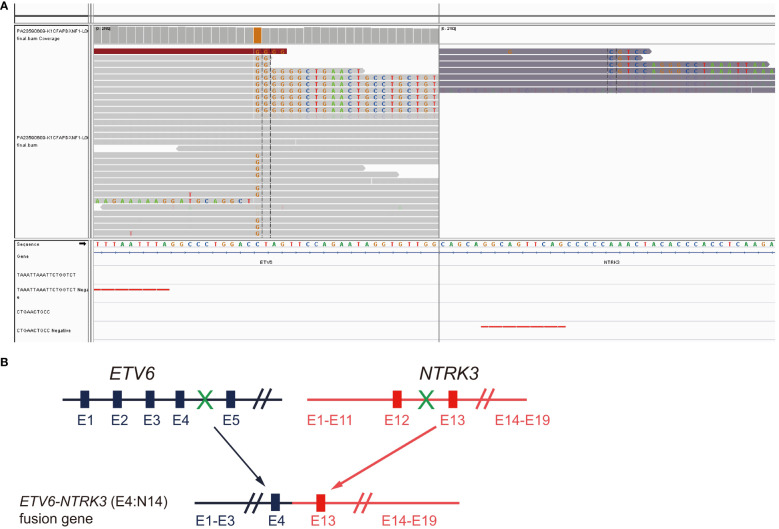
Detection of *ETV6-NTRK3* fusion following progression on fourth line brigatinib plus chemotherapy. **(A)** Integrative Genomics Viewer (IGV) snapshot of the fusion. **(B)** Diagram illustration of the chimeric protein generated.

A 56-year-old male former smoker (25 pack years) was diagnosed with lung adenocarcinoma (cT2N2M1c) with right pleural metastasis, effusion and left iliac bone metastasis in June 2019. His medical history was otherwise unremarkable, though it is noteworthy that his sister had been diagnosed with colorectal cancer. Genetic testing revealed the presence of an *EML4-ALK* (E20:A20) gene fusion. The patient was treated with frontline alectinib, achieving a best response of partial response (PR) (**Figure 1A**). In February 2021, computed tomography (CT) scans demonstrated the enlargement of pre-existing lesions, emergence of new lesions, and aggravated pleural effusion. According to the RECIST v1.1 criteria, these findings collectively indicate progressive disease (PD), with the patient achieving a progression-free survival (PFS) of 19 months (**Figure 1A**). After the initial progression, the patient underwent thoracentesis and began second-line treatment with ceritinib. Unfortunately, he experienced PD just two months later. Treatment was then switched to lorlatinib, which was associated with adverse effects, including persistent fever, aggravated pleural effusion, tumor growth, mediastinal lymph node enlargement, and pleural thickening. To manage these complications, the patients underwent thoracentesis for pleural empyema, pleural decortication, and cautery division of pleural adhesions. Then the patient was started on a combination therapy of lorlatinib with pemetrexed and carboplatin, which led to a best response of stable disease. However, the disease progressed again, with the detection of liver metastases in a CT scan in April 2022. Subsequently, the patient received six cycles of pemetrexed plus carboplatin and experienced PD five months later, as evidenced by new and enlarged liver metastases and new bone lesions on CT and bone scans. A pathological analysis of a liver biopsy verified the presence of metastatic adenocarcinoma (**Figures 1B, C**). The liver biopsy sample contains 50% normal liver tissue and 50% metastasized lung cancer tissue (**Supplementary Figure S1**). In the tumor tissue area, there is heavily filled with cancer cells, with only a small amount of connective tissue, and occasional lymphocytes and neutrophils are present. A smaller section of the sample displays an increase in fibrous tissue along with a higher count of lymphocytes, neutrophils, and a few eosinophils. The genetic profiles of the surgical samples and plasma were assessed using capture-based targeted deep sequencing, employing the GeneseeqPrime® panel with a sequencing depth of 3000X. This comprehensive panel analyzes the full exons, fusion-related introns, variable splicing regions, and specific microsatellite (MS) sites of 437 genes associated with cancer, spanning approximately 1.53 megabase pairs across the human genome (provided by Nanjing Geneseeq Technology Inc., China). This enhanced depth of sequencing facilitates a more accurate detection and characterization of genetic alterations, contributing to a deeper understanding of the cancer profile in each sample. A comparative analysis using the test results procured during diagnosis not only disclosed the persistence of the *EML4-ALK* (E20:A20) fusion that occurred with a mutation abundance of 35.38%, but also unveiled the emergence of the *ETV6-NTRK3* (E4:N14) fusion that appeared with a mutation abundance of 24.05%. In the following sections, we will provide a detailed discussion on the patient’s response to various treatments, as well as any adverse events that might occur for each treatment. There were no mutations in *EGFR*, *KRAS*, *ROS1* and *MET.* The tumor mutation burden (TMB) was 4.1 mutations/Mb and microsatellite status were stable (MSS). The variant frequencies of these fusions were 23.4% in the tumor tissue and 24.1% in the plasma, respectively, as shown in **Figure 2**. This newly identified *ETV6-NTRK3* (E4:N14), alongside the persistent *EML4-ALK* (E20:A20), might play a significant role in the observed resistance mechanism. Based on these findings, the patient initiated a fifth-line treatment regimen with brigatinib (90 mg d1-7 qd po followed by 180 mg qd po). Two weeks into brigatinib therapy, entrectinib was added to the regimen (400 mg d1-7 qd po followed by 300 mg qd po). A follow-up CT evaluation conducted two months after initiating this combination therapy revealed significant improvement in both pulmonary and liver lesions, indicative of a PR (**Figures 1B, C**). As of the most recent follow-up in May 2023, the patient’s disease has remained control, achieving a PFS of 8 months. Notably, genomic profiling on the plasma sample collected during the latest visit identified an *NTRK3* p.G623R mutation with a variant frequency 0.64%. This mutation may potentially be associated with resistance to entrectinib (6, 7), underscoring the importance of continuous genomic monitoring. The patient’s disease progressed, but he refused chemotherapy. Unfortunately, he passed away from liver failure in August 2023.

## Discussion

Long-term management of *ALK*-altered NSCLC poses significant challenges due to acquired resistance, necessitating the development of multiple generations of ALK TKIs to address ALK-dependent resistance mechanisms (8). However, overcoming ALK-independent resistance remains an ongoing challenge that requires further research and novel approaches. *NTRK3* is a member of the *NTRK* family of kinases, which are rare oncogenic driver genes in cancer, occurring at frequencies of 0.31% in adult tumors and 0.34% in pediatric tumors (9). In NSCLC, the most common partner of *NTRK3* fusion is *ETV6* (10). *NTRK* fusions are generally considered as mutually exclusive with *ALK* fusions, yet they have been identified as a resistance mechanism to EGFR TKI therapies (11). In the case report published by Garrido, *EML4-NTRK3* (E4:N14) fusion was detected after progression on lorlatinib. However, it remained inconclusive whether this fusion was acquired, as genetic testing had not been conducted prior to the initiation of ALK inhibitor treatment in that patient (5). Fortunately, in our case, genetic testing was performed both at baseline and upon progression on fourth-line treatment comprising lorlatinib, pemetrexed and carboplatin, which confirmed the *ETV6-NTRK* (E4:N14) fusion as a secondary event. However, the precise timing of the fusion’s emergence and the potential for earlier intervention remains unknown. Both of these cases highlight the significance of genomic profiling in re-biopsies for uncovering novel resistance mechanisms, and thereby facilitating timely and appropriate adjustments to the management strategy of the disease.

The differential response to entrectinib observed in our case and those reported in previous studies presents an intriguing aspect. In the previous reported case, entrectinib monotherapy was administered after progression on lorlatinib, yet it failed to elicit a clinical response. On the contrary, in our case, after observing the ineffectiveness of alectinib, ceritinib, and lorlatinib, a combination therapy incorporating both brigatinib and entrectinib was selected, which resulted in a notable clinical improvement. Several factors could account for the disparate outcomes between these two instances, including the potential superior efficacy of brigatinib over entrectinib in inhibiting EML4-ALK, the presence of entrectinib-resistant *ALK* mutations not detected in genetic testing in the reported case, or yet unknown resistance mechanisms. More research is warranted to reveal the underlying mechanism that led to the different outcomes in these two cases.

A final point to note in our study is the detection of *NTRK3* p.G623R mutation in the plasma eight months after the initiation of brigatinib plus entrectinib. This mutation was initially reported in patients with *ETV6-NTRK3* (E4:N14) fusion-positive patients manifesting secondary resistance to NTRK inhibitors (6). *NTRK* p.G623R is a solvent-front mutation homologous to *ALK* p.G1202R and *ROS1* p.G2032R mutations, all of which confer resistance to entrectinib (12). Hanf et al. reported response to cabozantinib following acquired entrectinib resistance in an *ETV6-NTRK3* (E4:N14) fusion-positive patient harboring *NTRK3* p.G623R (6). The significance of *NTRK* p.G623R on our patient’s clinical course and further treatment options awaits further follow-up.

## Conclusion

In summary, we report a case of *ALK*-rearranged NSCLC in which acquired *ETV6-NTRK3* (E4:N14) fusion was detected, and the patient derived positive clinical outcome to a combination treatment approach incorporating brigatinib plus entrectinib. Our findings provide clinical evidence supporting the role of *NTRK3* fusions in mediating acquired resistance to ALK inhibitor therapy and highlight the efficacy of combination therapy with ALK and NTRK inhibitors as a promising treatment option.

## Data availability statement

The original contributions presented in the study are included in the article/**Supplementary Material**. Further inquiries can be directed to the corresponding author.

## Ethics statement

This research was approved by the Ethics Committee of The Second Hospital of Dalian Medical University. Written informed consent to publish the clinical details and images were obtained from the patient.

## Author contributions

DL: Writing – review & editing, Data curation. YZ: Writing – review & editing, Methodology. JS: Writing – original draft, Resources. DY: Writing – original draft, Supervision. SC: Writing – review & editing, Investigation. XL: Writing – original draft, Visualization. LW: Writing – original draft, Project administration. JZ: Writing – original draft. EP: Writing – review & editing. ZD: Conceptualization, Writing – review & editing.

## Supplementary material

Supplementary Figure 1 H&E staining of the liver biopsy sample (**A** with magnification x100, **B** with magnification x200). The liver biopsy sample contains 50% normal liver tissue and 50% metastasized lung cancer tissue.
