# Supplementary figures and images for: Rapid response to fifth-line brigatinib plus entrectinib in an ALK-rearranged lung adenocarcinoma with an acquired ETV6-NTRK3 fusion: a case report
Source: Front Oncol. 2024 Apr 18;14:1339511. doi: 10.3389/fonc.2024.1339511 (PMC11063249; doi:10.3389/fonc.2024.1339511)

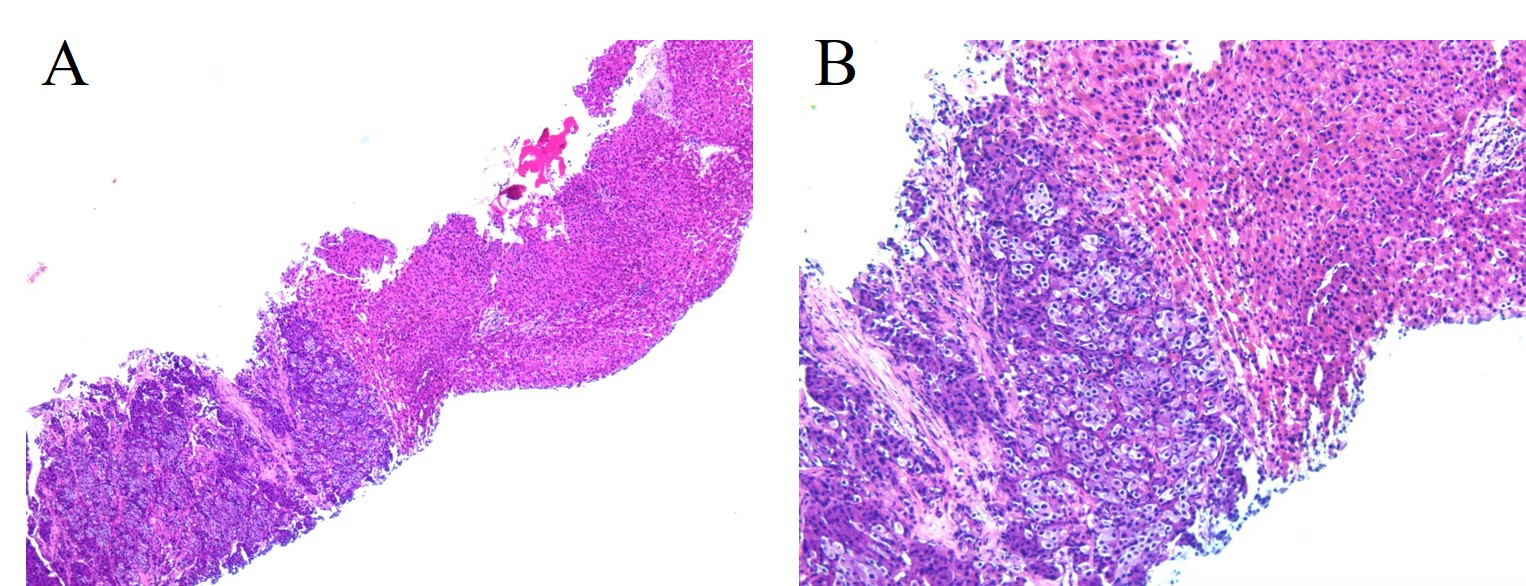

Supplement: Supplementary Figure 1 — H&E staining of the liver biopsy sample (A with magnification x100, B with magnification x200). The liver biopsy sample contains 50% normal liver tissue and 50% metastasized lung cancer tissue. [file Image_1.jpeg]
